# The aesthetic experience of the sublime for a group of Highly Sensitive Persons: Maselli's figurative style vs. Rothko's abstract expressionism

**DOI:** 10.3389/fpsyg.2025.1609994

**Published:** 2025-09-17

**Authors:** Fernando Echarri, Ignacio Miguéliz, Natalia Verea, Teresa Barrio

**Affiliations:** 1 University of Navarra Museum, Pamplona, Spain; 2 Education and Psychology Faculty, University of Navarra Museum, Pamplona, Spain

**Keywords:** sublime, contemporary art, contemplation, aesthetic experience, emotions, Highly Sensitive Person

## Abstract

Contemporary art museums have become important learning environments to promote visitor aesthetic education. Each piece of art constantly sends different messages to the viewer and creates a person-art connection that can provide significant experiences. These connections can be established in the contemplation of the sublime. In order to understand how these connections occur, researchers present a study about the relationship between aesthetic experience and the sublime that can happen through the contemplation of contemporary art, both figurative and abstract. Specifically, this aesthetic experience with the sublime has been studied in a group of highly sensitive individuals. The abstract work of Mark Rothko's masterpiece “*Untitled*” (1969) and the figurative work of Fernando Maselli “*Artificial Infinite*” (2014) have been utilized. The study includes an instrument for the evaluation of the “aesthetic experience of the Sublime,” in which four dimensions—perception, emotion, cognition, and spiritual—are considered. This instrument has been applied to a group of highly sensitive people. Based on mixed quantitative and qualitative data analysis, results show that these individuals can experience contemporary art painting intensely by perceiving changes in its sensitive features while vanishing self-references of time and space.

“*I believe that what can be taught in art is not fundamental; the fundamental things cannot be taught by anyone, you have to learn them yourself.”*

—Eduardo Chillida

## 1 Aesthetic experiences in art museums as meaningful experiences

One of the main purposes of art museums is to promote visitor aesthetic education (Falk and Dierking, 1992; Hein, 1998, 2000, 2012; Hooper-Greenhill, 2005; Falk, 2009; Burnham and Kai-Kee, 2011) and provide significant aesthetic experiences (Hein, 1998; Hooper-Greenhill, 2007; Filippoupoliti and Sylaiou, 2015; Kristinsdóttir, 2017). Museums can create and constitute a unique experiential place of variable forms (Falk, 2009), where visitors are encouraged to participate in aesthetic interpretation with their own creative skill. According to an experiential approach, the present research focuses on the challenge that museums face to foster the viewer's significant experience to artwork (Barnes and McPherson, 2019).

In connection with the above, the aim of this study is to analyze the relationship between the visitors' aesthetic experience and their engagement with the sublime through two different works of art, given that aesthetic and transcendent or spiritual experiences are intimately related (as it will be explained in more detail below). This research and its conclusions are based on a sample of individuals belonging to a group of Highly Sensitive Persons that, as discussed in Section 5, are characterized by a particular aesthetic sensitivity and a strong capacity for deriving deep and transcendent meanings from Art.

Visitor involvement in art contemplation is not a recent claim, as in 1925 the Spanish philosopher Ortega Gasset (2010, p. 310) had already argued for the need of pause, detention and slow contemplation in art interpretation vs. quick glazing and passing by the art object. Contemplation is a gate to experience. “We must teach observation” César Manrique used to say (Gómez, 1990). It is through the education of our sight that we can reach the “communication with objects” (Yenawine, 2013, p. 39) and capture possible messages. Art experience requires visual dialogue between viewer and artwork at the deepest levels of insight, in which first sensitive arousal evolves progressively into emotional, cognitive and meta-cognitive response, ultimately becoming what Pelowski and Akiba (2011) call “transformative aesthetic experience.”

In order to foster this kind of experience a museum facilitator faces a challenge similar to that of the artist when trying to communicate immaterial experience through material form. Facilitators must design proposals according to viewer immaterial features and object material features, since both, subject and object, together determine educational experience as well as aesthetic experience, in the sense that these two experiences mean novelty and genuine human growth (Dewey, 1934). However, strong experiences like transformative aesthetic experiences always emerge in a particular, intimate, unexpected way for each human being, meaning the educator never has guarantee or total control over it and only approximation is possible, by means of designing, planning, and implementing best conditions for it to succeed.

Previous research has addressed the use of art to generate an experience of the Sublime. The study of Stamatopoulou et al. (2024) is a solid example that exposes the interplay of the aesthetic and the spiritual, using religious paintings and exploring the reaction of a range of participants to them. The question is whether other forms and subjects of art, beyond strictly religious ones, may serve as appropriate sublime stimuli to foster a similar experience to the one provoked by religious art, a possibility already suggested by the aforementioned authors. Fingerhut and Prinz (2018, p. 122–123) also raise the question of whether any artistic genre can elicit awe (intimately connected to the Sublime experience), an issue that we seek to address through a case study in this article.

## 2 Personal dimensions of art contemplation

Aesthetic education bases on the idea that human life is sensitive to the qualities of the material world and to how these qualities relate to each other (Eisner, 2002). Since art is about the mastery of relating qualities, its contemplation offers the best opportunities for aesthetic education. Art sensibility has to do with material physical perception as well as immaterial psychological perception, as arousal connects with emotion and cognition making. For this reason, it is said that art can profoundly touch and change lives.

According to the model of “transformative aesthetic experience” of Pelowski and Akiba (2011), true art contemplation is a complex phenomenon that goes beyond assimilating art information. It is neither self-escape nor any other kind of evasion from the real world. On the contrary, it demands overcoming initial cognitive disruption and discrepancy, by deeply touching the inner self (Vining and Merrick, 2012).

The aesthetic experience may be essential for a deeper understanding of the world around us and of ourselves. Effectively, art is not merely an aesthetic object to be enjoyed, contemplated, or received in a subjective and individual manner, isolated from life and knowledge. Rather, it constitutes a mode of understanding and an encounter with truth and with the world. Art is a manifestation of truth in an existential or revelatory sense (Gadamer, 1989).

Teaching and learning to contemplate an art object requires attention to different dimensions of the person that integrate together to produce what is called the aesthetic experience. In this regard, we can say that “There is much more body and much more soul to be rendered if you have the will” (Bumgarner, 2004). Fingerhut and Prinz (2018) refer to intense and complex engagements triggered by the emotion of wonder, in which cognitive, perceptual, and spiritual components can be identified, and, when sufficiently strong, may culminate in an experience of awe. Bearing this in mind, we refer to the body dimensions: sensitive, emotional, cognitive, and spiritual.

## 3 Art contemplation and the sublime concept

The concept of the Sublime first appears in an anonymous work attributed to Longinus from the 1st century AD, *On the Sublime*, which approaches it from the perspective of literary genres rather than aesthetics. In one of its chapters, an analysis of the figure of the Sublime is included in relation to the vastness of the cosmos, though linked to humanity and not the external world. In 1756, the British philosopher Edmund Burke (2017 [orig. 1756]) revisits this idea. He conducts an empirical analysis of aesthetic terms that remain undefined. Burke focuses on identifying and differentiating the Beautiful and the Sublime, which he sees as qualities that are recognizable in objects. He defines the Sublime as a controlled fear that attracts the soul, present in qualities such as vastness, infinity, emptiness, solitude, or silence. According to Burke, nature has the capacity to provoke extreme emotional states in the subject, introspectively stirring their deepest “self,” feeling in that moment both ecstasy and anguish. In contrast to the Beautiful, the Sublime escapes the domains of reason and fully enters the realm of feelings and senses, of the irrational.

A few years later, the German philosopher Immanuel Kant (2000 [orig. 1790]) analyzes these same concepts of the Beautiful and Sublime. For him [Burke (2017 [orig. 1756])], the Sublime is “that whose mere thought gives proof of a faculty of the mind that exceeds all measure of the senses.” Kant differentiates between the Sublime, which involves experiencing feelings that cannot be explained through reason, and the Beautiful, which he understands as feelings that can be comprehended through intellectual capacity.

For both Burke and Kant, a fundamental component of the Sublime, which Kant links to what he calls the Terrifying Sublime, relates to feelings of horror, melancholy, nostalgia, sadness, or solitude, acquiring a certain negative character. For them, fear is an indispensable feeling if one wishes to experience the Sublime, so solitude is seen as a path to confront this concept. For these authors, the Sublime entails the exaltation of the viewer's senses, requiring emotional involvement with what is contemplated, far from a state of indifference. It is connected to the ability to feel sensory experiences when contemplating the deepest beauty, but at its most extreme and irrational degree –ecstasy- so that this experience can even cause pain due to the difficulty of fully assimilating it, provoking in the viewer a state of excessive sensory disturbance. Therefore, the experience of the Sublime produces the strongest emotions that the spirit is capable of feeling.

More recent authors offer valuable insights into the Sublime. According to Skorin-Kapov (2016), the Sublime has different consequences in the realms of aesthetics and ethics: in aesthetics, it invites reflection, astonishment, or admiration, while in ethics, it demands a response, a movement toward action or moral commitment. Despite these differences, the Sublime remains a major foundation for both ethics and aesthetics. Sublime art experiences can begin as a simple awe response or “thin sublime” but through extended self-reflection, they can evolve into a “thick sublime,” a deeper, transcendent experience that evokes reverence. This self-reflection is key to transforming an awe experience into a truly transcendent one (Stamatopoulou et al., 2024).

For his part, Clewis (2019) contends that the philosophical notion of “sublimity” may be identified with “aesthetic awe” within psychological discourse, a view in line with Fingerhut and Prinz (2018), who maintain that awe plays a central role in facilitating the aesthetic experience of the sublime. As explained by authors such as Guan et al. (2019) it is possible to distinguish between positive and negative awe. The former is associated with calm states and a tonic positive affect, along with higher appraisals of personal control over the situation. The latter, by contrast, is linked to feelings of fear and powerlessness, as well as reduced self-control and a diminished sense of certainty. As a consequence, the sublime can be described as a feeling or experience that combines elements of exhilaration and discomfort, grounded in both positive and negative awe and capable of provoking a wide range of responses in the viewer.

In a similar vein, Konečni (2011) develops the Aesthetic Trinity Theory, in which a tripartite hierarchy of responses is proposed, with aesthetic awe as the peak experience and tightly associated with the sublime. This experience is rare and powerful, particularly intense, deep, and memorable. Joy and fear arise, although the experiencing person enjoys existential security in front of the sublime stimulus, characterized by physical magnitude, rarity or novelty.

In this historical and theoretical context, Art has attempted to represent the concept of the Sublime on numerous occasions, with perhaps its main reference being the Romantic work “The Wanderer above the Sea of Fog” (1818) by the German artist Caspar David Friedrich. In this work, one can explicitly appreciate a person facing the vastness of nature as seen from the top of a mountain. In contemporary times, the concept of the Sublime can be represented in a figurative style by artists like Fernando Maselli, or in an abstract style, as can be seen in the aesthetic proposals of Mark Rothko. Both proposals are described in the following section.

## 4 Different artistic styles that can generate aesthetic experiences of the sublime

### 4.1 Overview

This article suggests that self-reflectivity and a significant experience linked to the sublime can be achieved through both abstract and figurative art, not necessarily through religious themes. To that end, the selected artists were Mark Rothko and Fernando Maselli, respectively. Rothko's aesthetic proposal is a form of abstract art strongly grounded in the power of color fields; in Maselli's case, the chosen approach is the representation of nature in all its magnificence.

In the work of both artists, it is possible to find an underlying and intentional “*religious experience.”* This is evident in a paradigmatic work by Rothko: the so-called *Rothko Chapel* in Houston, which exemplifies this dimension perfectly but also reflects a thread running throughout the artist's oeuvre. In the case of Maselli, projects such as *Annunciation* (Maselli, 2025a) or *Hierophanies* (Maselli, 2025b) appear explicitly, yet the transcendent is also patent in the natural landscapes of *Artificial Infinite* (Maselli, 2017). In Rothko and Maselli the search for the spiritual is consistently present in both formal and thematic choices—not only in the subject matter, but also in the composition, perspective, symbolism, and use or absence of colors.

Rothko's art aims to envelop the viewer, provoking a sensation of “being inside the painting” through the scale of his works and the atmospheric quality of his forms and colors. Abstraction may be considered particularly suited to expressing the ineffable (i.e., the sublime) by alternative means.

Maselli, by contrast, confronts the viewer with the grandeur of untamed nature. Since both art and nature provide the appropriate experiential context for a sublime aesthetic experience (Skorin-Kapov, 2016), Maselli's combination of the two seems to offer an excellent and accessible vehicle for achieving this aim.

### 4.2 The figurative style of Fernando Maselli: the creation of the “*Artificial Infinite*”

Fernando Maselli (Buenos Aires, 1978) is an Argentine photographer residing in Madrid, whose work reflects pristine natural spaces, conveying his interest in aesthetic concepts such as the Sublime. Through his photographs, he addresses the relationship between human beings and nature, explaining vital notions for humanity such as spirituality.

His works recreate sublime landscapes generated through digital collages that juxtapose real images to form new views that emphasize the majesty of nature. In his work, Maselli seeks to confront the viewer with nature and evoke feelings that lead to the perception of the Sublime. In this series, Maselli represents not only what is seen but also what is felt. As he defines it, *Artificial Infinite* (Maselli, 2017) is “a photographic investigation into the aesthetic state of the Sublime, represented as a controlled fear that attracts the soul […]. The sublime is often associated with qualities such as immensity, infinity, emptiness, solitude, or silence.” (To view this work, please visit: https://coleccionmun.unav.edu/objects/78348/monte-patterno?ctx=a5258d0ede47ef7a1d5e2319f3ae71438c415f2a&idx=4). In its approach to the sublime, through solitary experience of the majesty of nature, Maselli glimpses the abyss of infinity and emptiness, just as Carpar Friedrich did in his paintings. It seeks for the persons to be able to feel their smallness, are overwhelmed by the immensity of nature and feel the Sublime.

### 4.3 Abstract expressionism and the generation of the Rothko experience

Mark Rothko was born in Dinsk (Latvia), in 1903. He moved to the United States with his family at age ten and settled in Portland, but moved to New York City in 1923. By 1958, he began to paint his famous “multiforms,” where he applies his notable “color abstraction” (Kandel, 2016, p. 123). His style includes “color fields” that generate a “sense of space and light on the canvas” (Kandel, 2016, p. 128). His color fields allow for a journey that begins in perception and can advance toward emotional, cognitive, and spiritual spheres. (Kandel 2016, p. 126) thinks that “Rothko's work became most complex and challenging when it appeared simplest.” Rothko's language is highly simplified but “despite the apparent simplicity of the shapes […] was extremely exacting and precise in conception and invention” (Arnault, 2023, p. 2). He uses a reductionist visual vocabulary focused on color and depth, creating a special beauty.

In general, and concise terms, we could say that these multiforms often use rectangles (generally two, sometimes one or three) of evanescent color that seem to float over a colored background. It could be said that it builds “towering, luminous abstract paintings” (Rothko, 2023, foreafter). Their edges are diffuse, and the uninterrupted colors maintain a certain discontinuous heterogeneity. From these colors emerge different luminosities that coexist between light and shadow. Therefore, in his works, you see nothing in the sense that there are no concrete objects in his rectangles and backgrounds. However, his works provide windows into a world of infinite possibilities, where anything is possible. In his color fields, there is nothing, but everything is there.

His work enables those activities of “meditational look” (Muñoz, 2008) because through his “color fields” (Chave, 1989), he can create “an intimate and human relation between painting and viewer” (Christensen, 2017). These peculiarities of his painting allow “our brain to form new ideas, associations, and relationships—and new emotional responses to them” (Kandel, 2016, p. 128). This perceptual-rational-emotional mechanism enables the generation of aesthetic experiences that can create engagement, a bond with the viewer. Rothko reaffirms this, for whom his painting equates to an experience: “a painting is not a picture of an experience. It is an experience” Kandel, (2016, p. 130). (Phillips and Crow 2005) call this experience the “Rothko experience.” It is an experience where the “I” does not explain the experience, but the experience explains the “I.” That is why Rothko paintings can be used for contemplation of the Sublime.

### 4.4 Figurative art vs. abstract art

Possibly, there are many artistic languages that can be used for the contemplation of the Sublime. When referring to the confrontation between figurative and abstract styles, it is important to highlight some aspects. In general, abstract language presents more difficulty than figurative language when it comes to interpretation. Our mind inevitably seeks to find recognizable aspects in a work of art. This happens more easily in a figurative work than in an abstract one. Therefore, one might think that the viewer's perception of the Sublime is more readily achieved in Maselli's photographs than in Rothko's painting. In the former, the viewer directly confronts the Sublime landscape constructed by Maselli. In this sense, it can be said to be easily recognizable.

In contrast, in Rothko's work, the viewer stands before an abstract painting that initially prevent the viewer from finding recognizable objects, beyond the rectangles. This compels them to confront themselves, as Didi-Huberman (2006) indicates: “each work interacts with the viewer according to their own background, responding sensorially to the stimulus that the work provokes in us.” The work becomes a challenge. To unravel this challenge, the work must be activated. In this way, Maselli's work explicitly, directly, and tangibly presents the image of the Sublime to the viewer, while Rothko's demands an exercise of introspection and effort on our part to discover the Sublime that lies hidden within it. All of these ideas are connected to our third hypothesis, presented in Section 6.

## 5 Aesthetic sensitivity in highly sensitive people

Although all individuals are sensitive, it has been shown that some are more sensitive than others and tend to be classified into three different groups along the sensitivity spectrum: approximately 30% are classified as having low sensitivity, 40% as having medium sensitivity, and 30% as having high sensitivity. Researchers define highly sensitive individuals as those who are more affected by what they experience. This can include how they are influenced by the physical environment, as well as by social relationships, work conditions, and education, among other factors (Pluess et al., 2023).

To describe these differences in sensitivity, several psychological theories have been developed. Among the main ones are: the Differential Susceptibility Theory, the Biological Sensitivity to Context theory, and the Sensory Processing Sensitivity (SPS) theory, which was introduced by psychologist (Aron 1997) to describe the group of individuals with high SPS, who would later be referred to as Highly Sensitive Persons (foreafter HSP) in 1996. They are characterized by greater awareness and responsiveness to environmental stimuli, deep processing of information, heightened emotional reactivity to stimuli, and an awareness of subtle details in their surroundings (Aron and Aron, 2016; Acevedo et al., 2018).

Research by Smolewska et al. (2006), Chacón et al. (2021), and De Gucht et al. (2023) identified three underlying dimensions of SPS and distinctive factors of high SPS: ease of arousal (EOE), low sensory threshold (LST), and sensory and aesthetic sensitivity (AES), characterized by a strong interest in art, intense emotional appreciation of beauty, and notable creative potential (Smolewska et al., 2006; Bröhl and Schury, 2020; Khosravani et al., 2021; De Gucht et al., 2023). Additionally, Chacón et al. (2021) identified fine psychophysiological discrimination (FPD) and harm avoidance (HA).

In behavior, the depth of information processing manifests as good memory, awareness of subtleties, connection of ideas, active listening, recognition of themes, and meaning-making (Bridges and Schendan, 2019). HSP are especially equipped to grasp spiritual language. As Aron (1997) states, “in Highly Sensitive Persons, there is something that is more soulful and spiritual.” There are aspects of this personality trait that favor it. For example, their need to extract deep meanings from events, the ability to understand and use symbols, their orientation toward helping others, their empathy for the suffering of others, and their hunger for integrity and honesty (Pardo, 2018).

In addition to being more receptive to stimuli and processing information more deeply, highly sensitive individuals, compared to less sensitive ones, feel intensely and perceive images and sounds in an amplified way. In the brain, this is observed as greater activation in areas responsible for complex and integrative processing of multisensory modalities and higher-order mental processes that demand cognition (Acevedo et al., 2018).

In an fMRI (functional Magnetic Resonance Imaging) experiment, it was observed that “individuals with high sensitivity showed greater brain activation […] in the functional areas of the brain involved in visual attention and eye processing.” They demonstrated “an ability to notice subtle changes” in images, which is due to the fact that “they process sensory information more elaborately than individuals with low sensitivity, meaning they have greater attention to detail and nuances” (Pardo, 2018).

Using the same technique, fMRI also demonstrated that highly sensitive individuals have a different empathic circuit. This manifests in responses to positive stimuli (the face of a loved one, the beauty of a landscape) that in highly sensitive individuals are associated with greater activation of the reward system, compared to less sensitive individuals (Acevedo et al., 2014).

On the other hand, the perception of HSP is less influenced by others and by the cultural context of the stimulus than that of less sensitive individuals. Being more attentive to all aspects of stimuli makes them less sensitive to the perception of cultural differences, freeing them from stereotypes and prejudices. Their perceptual model provides them with freer reflection and enhances their abilities to perceive all characteristics of a stimulus, beyond its cultural meaning, favoring their skills to perceive artistic language (Aron, 1997; Pardo, 2018).

Studies have also found that high sensory sensitivity is associated with negative outcomes such as low mood, stress, burnout syndrome, introversion and inhibition, anxiety, shyness, and depression (Aron et al., 2012; Karaca Dinç et al., 2021).

From this general metaframework arises the concept of Environmental Sensitivity, which explains that individuals differ in their sensitivity due to individual differences in their ability to perceive and process information about the environment.

## 6 Design of the study

This study bases on three fundamental research questions that build its theoretical framework from a general to a particular approach. Specifically, based on some indicators of aesthetic experience, the study examines how the contemplation of the works impacts four dimensions of human development -perceptual, emotional, cognitive, and transcendent- in order to approach the potential of the “Aesthetic Experience of the Sublime” in HSP. Under this intention, we pose the following research questions (RQ):

RQ1. The contemplation of Fernando Maselli's and Mark Rothko's artworks arouses aesthetic experience of the Sublime in the HSP?
RQ2. The aesthetic experience of the Sublime reaches the perceptual, emotional, cognitive and even spiritual dimensions of the person in HSP?
RQ3. HSP can link with Sublime through figurative artwork made by Maselli more easily than abstract artwork made by Rothko?

Our initial hypothesis regarding these RQ are:

H1. The contemplation of artworks by Fernando Maselli and Mark Rothko can provoke a significant aesthetic experience of the Sublime for HSP.
H2. The Sublime Experience Scale has a factorial structure that encompasses four dimensions (perceptual, emotional, cognitive and spiritual) that, when integrated, show the intensity of the aesthetic experience with the Sublime. One of our references in this respect is Fingerhut and Prinz (2018).
H3. Contemplating the Sublime, figurative art of Maselli affects the different spheres of a person more significantly than the abstract art of Rothko, and also the experience of the Sublime is better.

This hypothesis is based on studies that use religious figurative art to prove the connection between the aesthetic and the spiritual (Stamatopoulou et al., 2024). As pointed out in Section 4.4., there is a common understanding that figurative art is more easily appreciated by the majority of people, as traditional or conventional artistic languages make it easier to identify and recognize value in the subject matter, the accuracy of drawing, composition, perspective or the use of color, other than the fidelity to the reality being represented. The most recent developments in Art History are often misunderstood or even rejected (Echarri and Urpí, 2022, p. 10–14).

### 6.1 Sample

HSP is the target group for this case study, as stated in Section 5. Specifically, 27 individuals were selected to participate in the study as good informants by the delegate in Navarra of the Spanish Association of Sensitive Education (SASE) among the members of this entity who have regularly attended activities organized under the Sociarte Project -an initiative of University of Navarra Museum aimed at bringing art closer to groups from different non-profit associations in Navarra-. Specifically, SASE directs its activities toward the group of highly sensitive people (HSP). Participation in the Sociarte Project guarantees an appreciation for the art, the habit of contemplating works of art, and the aesthetic education of the participants in the study. Additionally, they verified their high sensitivity through the application of the Highly Sensitive Persons Scale (HSPS) in its version adapted for the Spanish adult population by Chacón et al. (2021). This tool consists of 27 items grouped into five dimensions, among which is aesthetic sensitivity (AES). The other four dimensions are: sensitivity to overstimulation (SOS), low sensory threshold (LST), fine psychophysiological discrimination (DFM), and harm avoidance (HA).

### 6.2 Study context

All the activities took place in one of the exhibition rooms of the University of Navarra Museum, where the selected artworks were displayed. The participants were Highly Sensitive Persons (HSP) and the goal was to promote a meaningful experience on the Sublime mainly through the contemplation of two artworks, “*Artificial Infinite*” by the artist Fernando Maselli, and *Untitled* (Rothko, 1969) by the artist Mark Rothko (to view this work, please visit: https://coleccionmun.unav.edu/objects/70486/untitled?ctx=c16b12ff3658f011cadc9a3667ed2f5878436f26&idx=0). The activity was conducted by a museum facilitator using the “Visual Thinking” methodology, which included a phase of long-term contemplation in silence in the gallery, in front of the artworks (15 min each artwork), followed by a collective and dialogic activity based on the contemplation, through several open-ended questions asked by the museum facilitator (Arnheim, 1969; Yenawine, 2013). Firstly, the artwork by Maselli was contemplated and discussed; the contemplation and discussion of Rothko's painting followed. All the comments were recorded in audio format and, finally, participants completed the “Sublime scale” questionnaires related to both artworks, provided and collected always by the same facilitator.

### 6.3 Instrument proposal

To evaluate the experience, the Scale for Evaluating Aesthetic Experience developed by Urpí et al. (2022), which has undergone preliminary validation, was adapted to include items more related to the attributes of the concept of the Sublime gathered from the literature. That is why it was called the “Sublime scale.” The adaptation of this measurement tool was debated and jointly agreed upon by the study's researchers, with an awareness of the limitations involved in gathering reliable data based on participants' self-reports (Fingerhut and Prinz, 2018, p. 122).

The Sublime scale instrument collects data related to perceptual, emotional, cognitive, transcendental, and experiential spheres, organized to evaluate the impact of the experience in a comprehensive manner on the corresponding psychoeducational areas of contemplating the artwork. Each area was developed with numbered items through Likert-type questions containing values ranging from 0 to 5 (Table 1). After the Likert questions, the questionnaire asks a general question about the satisfaction with the overall experience. This question (number 6: Could you rate your satisfaction with the experience of contemplating the artwork from 0 to 10?) has a measurement range from 0 to 10. Moreover, the following open-ended question was included at the end of the questionnaire in order to collect qualitative data: “Could you describe in your own words what your experience of contemplating the artwork was like? Feel free to write as much as you need.”

**Table 1:** Means of the items on the measurement scale closest to the concept of the Sublime.

**Code**	**Item**	**Maselli**	**Rothko**
P9	Subtlety	4.4	4.1
E1	Astonishment	3.8	3.3
E2	Fear	3.1	2.6
E3	Joy	2.8	3.3
E11	Unease	3.1	3.1
E15	Admiration	4.3	3.7
E19	Immersion in the artwork	4.3	3.8
C10	Infinity	4.2	3.0
C11	Unattainable	3.8	2.5
C14	Material beauty	3.4	2.7
C15	Sublime	4.3	3.3
R4	Sense of life	3.9	3.1
R5	My existence	3.8	3.4
R8	Transcendent beauty	4.1	3.1

The reader may find the complete Sublime scale as Appendix 1.

### 6.4 Ethical considerations

Ethical approval for the study, along with the consent form and questionnaires, was granted by the University of Navarra Ethics Committee (reference number: project 2019.159; approval date: 10/24/2019). Prior to data collection, participants were informed about the voluntary and anonymous nature of their participation in the study. Lastly, all participants were provided with an easy-to-read informed consent form in Spanish to sign. The University of Navarra Ethics Committee confirmed that the information and consent process complied with the legal requirements of Spanish law. Research transparency is a core principle of this study. Consequently, all activities related to the design, planning, and implementation of the study have been conducted in compliance with the Spanish Protection of Personal Data and Digital Rights Act (LOPD) 3/2018. The content management tool provided by the University of Navarra was used to ensure the ethical collection and storage of all research data, in accordance with the European Data Protection Regulation (GDPR 2016/679). Finally, all personal data were processed anonymously and confidentially, following the globally recognized ethical standards of the American Educational Research Association (2011).

## 7 Results

The quantitative and qualitative results obtained from the application of the Sublime scale, with questions, are shown below, taking into account the research question and hypothesis posed in the research. Of the 27 participants, 27 answered the scale.

### 7.1 Quantitative results

The results were obtained from the Sublime scale, with Likert-type questions containing values ranging from 0 to 5. Regarding our hypothesis HP1, the results obtained were measured in the questionnaire on a scale from 0 to 10 about the level of satisfaction with the experience (Question 6). In the case of Maselli, it was 8.3 out of 10, while in the case of Rothko was 8.4.

**Figure 1:**
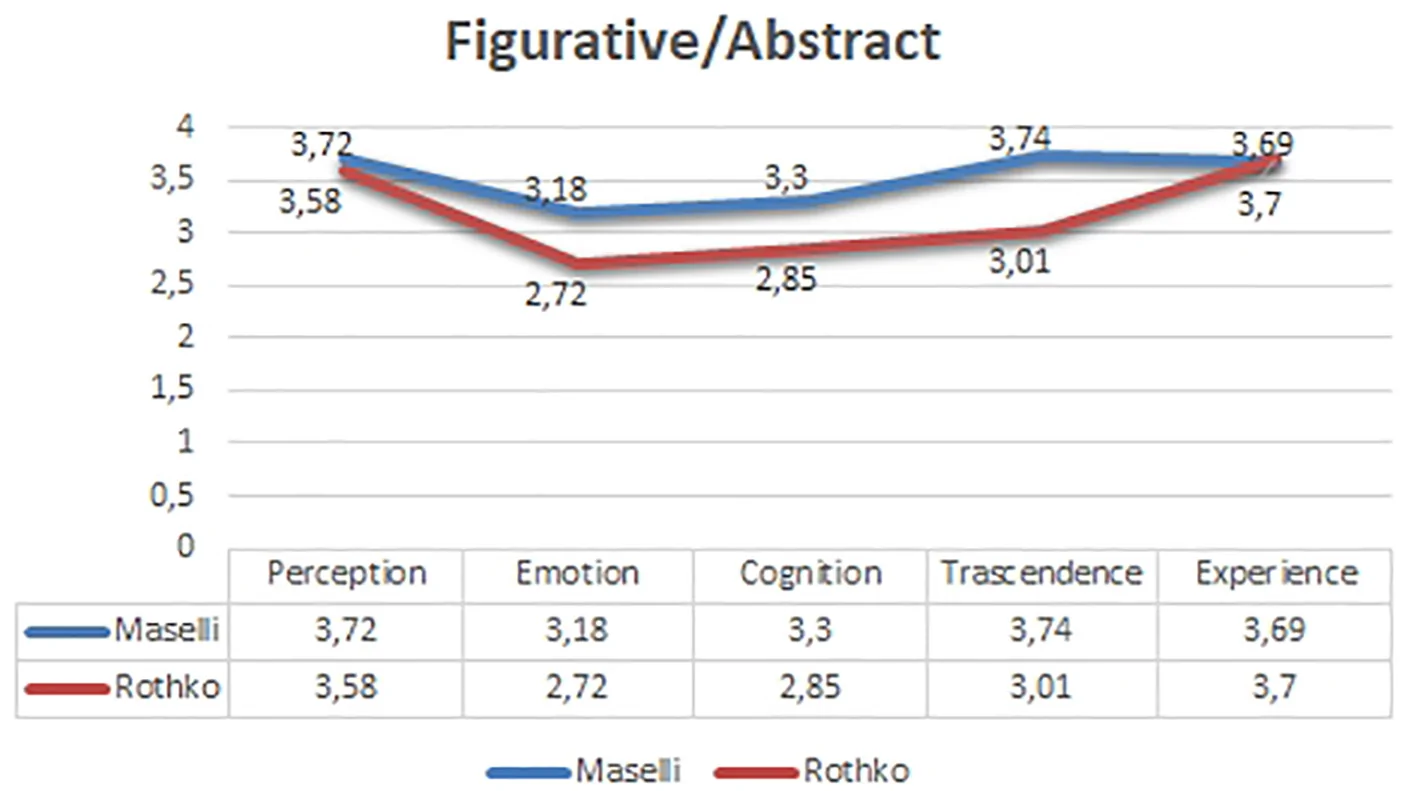
Values of dimensions for abstract and figurative artworks.

Regarding the hypothesis HP2, Figure 1 shows that figurative artwork obtained higher values that abstract painting in all categories: Perception (3.72 vs. 3.58), Emotion (3.18 vs. 2.72), Cognition (3.30 vs. 2.85), Transcendence (3.74 vs. 3.01).

Regarding the hypothesis HP3, in Experience field where abstract is higher than figurative (3.69 vs. 3.70); the difference found using Student's *t*-test was significant in all categories: Perception [*t*_(26)_ = 0.6, *p* = 0.47, Cohen's *d* = 0.009]; Emotion [*t*_(26)_ = 0.006, *p* = 0.69, Cohen's *d* = 0.51]; Cognition [*t*_(26)_ = 0.09, *p* = 0.07, Cohen's *d* = 0.64]; Transcendence [*t*_(26)_ = 0.002, *p* = 0.033, Cohen's *d* = 1.86]; Experience [*t*_(26)_ = 0.89, *p* = 0.07, Cohen's *d* = −0.06].

The results obtained from the items most directly related to the concept of the Sublime are shown in Table 1, in the form of means.

### 7.2 Qualitative results

Following the model of Miles and Huberman (1994), an open, axial, and flexible coding procedure was used for qualitative analysis, consisting of moving from raw data to a system that represents them through the assignment of codes. To carry out this process, inductive reasoning was employed, through which the codes were extracted from the data via continuous reviews. The content analysis technique was used for exploratory purposes, along with the search for correspondence with the theoretical framework through the identification of perceptions and meanings present in the text in relation to the specific objectives of the scale questions.

The results from the open-ended questions of the “Sublime Experience Scale” confirm that both the contemplation of the works of Maselli and Rothko evokes an aesthetic experience of the Sublime, and that this reaches the perceptual, cognitive, emotional, and transcendent-spiritual dimensions of the person.

#### 7.2.1. H1: Aesthetic experience of the sublime

Regarding hypothesis H1, in the case of Maselli, experiential testimonies appear about the experience of the sublime: “*It is beautiful*” (M11, M10); “*Despite being very rocky and steep, I felt incredibly good in the place*” (M18); “*I liked it a lot*” (M20); “*It is admirable for its sublimity*” (M22); “*The power of seeing a sublime landscape has allowed me to access its mysteries and empathize with it*” (M26).

In many testimonies, concepts associated with the sublime appear, such as. embodied in concepts such as wonder: “*It seemed so real that I felt I could hear the wind and feel it on my face, […] I sat with my legs hanging off one of those mountains to admire that tremendous wonder*” (M10); “*It has been a wonderful experience of inner connection*” (R17); inmensity: “*[…] to immerse myself among the mountains and live the peace of knowing your smallness*” (M24); enjoyment: “*I have never had an experience like this in my life, and I have been to many museums […] and I say: ah, what a delight!*” (M3); admiration: “*I liked it a lot for its drama […] I am fascinated by it*” (M1); anguish and anxiety: “*I felt a lot of anguish seeing my smallness reflected against the vastness […] I felt a kind of anxiety*” (M6).

Another way of perceiving the sublime emerged through the contrast between negative and positive feelings: “*I felt nostalgia, but at the same time peace, beauty, awe*” (M10); “*I perceived the yin (tranquility, meditation, silence, peace, beauty, infinity, light, protection, calm) and the yang (alertness, vastness, helplessness, cold, desolation, fear, darkness, survival, wind, nature)*” (M11).

In the case of Rothko there are testimonies indicating the experience of the sublime: “*I enjoyed contemplating this painting a lot*” (R6); “*The truth is that I liked this one much more, it has given me much more to think about*” (R8); “*It has been a tremendous marvel […]. I want to take it home and have a cup of tea with cookies while I delight in contemplating it*” (R11); “*I have achieved a friendship with it*” (R14); “*I loved it*” (R19); “*[It helped me] to focus on the sublime and confront that immense world through emotion, thought, and transcendence*” (R26).

There are also those who deny the sublime in the work (R1), in contrast to those who assert that the experience of the sublime in Rothko surpasses that in Maselli: “*[…] this experience has been much more sublime than the previous one, because the work spoke to me without me asking for it, it revealed itself to me unexpectedly and has changed for me. It has been a gift*” (R25).

Explicit references to the sublime are less frequent than in Maselli case and express opposing impressions. Some experience a lack of life where others find vitality: “It conveys the idea of that vitality, of that stridency” (R1); “[It conveys to me] a sense of immensity and also desolation. A lack of life. Something inhospitable, barely habitable, but admirable for its sublimity” (R22).

#### 7.2.2. H2: Intensity of experience in four personal dimensions

##### 7.2.2.1 Perceptual dimension

In the contemplation of Maselli, external stimuli were activated, mainly related to temperature: “*I felt the warmth of the sun, but I felt the cold of the snowy landscape*” (M6); “*[I have felt] a lot of heat*” (M8); “*[I have felt] the heat of the desert and the cold of the mountain*” (M14); “*I felt cold*” (M19, M23); also with smell: “*I liked that smell of winter and nature*” (M19) and hearing: “*I felt silence and sound*” (M14). Participants captured nuances that gradually intensified: “*I always stopped in the distance because I didn't even see the distant mountains the first time. And then, my eyes kept going back there to the background, and I even saw more colors each time*” (M3), “[*I have seen] nuances, details, levels of complexity (…) strata*” (M7).

In the case of Rothko, the recorded external stimuli referred to temperature: “*It feels warm, the heat, the joy, it conveys a lot to me*” (R4), “*It evokes warmth for me*” (R11), “*It inspires warmth and evokes summer and the memories it generates*” (R13), “*At first impression, it was warmth, it overwhelmed me, it transmits too much heat*” (R14), and to taste: “*The first impression I had when sitting down is that I want to suck it! […]*” (R11).

And just like in the contemplation of Maselli, participants captured nuances in Rothko's work that gradually intensified: “*[…] being able to contemplate it so closely helps me understand the elegance of the master, how to make a technically complex work seem so easy”* (R11); “*I struggled to connect with it […] Until suddenly I started to see dark colors that caught my attention […] there is more depth behind what we see*” (R15); “*This work, seen very closely, made me see the subtleties of the textures, where I previously only saw colors*” (R24); “*When looking closely, the eye changes or reinterprets the shapes or colors or sensations*” (R23).

##### 7.2.2.2 Cognitive dimension

In some participants, unconscious internal stimuli were activated that allowed them to perceive immaterial aspects in the material work of Maselli, touching their deep selves: “*I have felt the masculine and the feminine, the attainable and the unattainable (…) I have seen the sky and spiritual peace and hell and restlessness, […] the self and the you*” (M14); “*It has been like living a process of overcoming stages of my life, a journey to the past to understand where I come from and everything I have achieved in terms of personal growth*” (M17); “*It reminds me that if I put myself there, what my own size is*” (M24).

A new level of vision, which reaches internal awareness and is achieved through depth in contemplation, is also reflected in the communication with the objects of Maselli's work: “*[…] Then there has been a beautiful experience of listening to them. And they spoke to me of the time of origin, of many years ago […] the ephemeral nature of my life, but of the opportunity to live experiences of fullness, connection, and beauty that reveals itself to us*” (M15); “*It gives me the impression that the artwork is speaking to me: you need to change your life*” (M22).

In the case of Rothko's work, the more unconscious internal stimuli are also abundant: “*It conveys the idea to me […] that we do not find everything done, everything defined, everything thought out […] But it conveys the idea of that vital flow, or that vital movement as continuous”* (R1); “*It has invited me to dialogue as a couple, I have realized […] how art and its contemplation help us to be more human*” (R3); “*I have seen how they give a voice to silence, a throat with which to speak and how the despair of being alone drives us mad*” (R9); “*I see the volcano, I see the war in Ukraine, I see passion, I see the maternal womb […] “It has served me to […] open up to my area of discomfort*” (R24); “*[…] what I see is like burning, fire, even extermination, I mean, it's too much, for me it's too much*” (R25).

Some participants mention difficulties in deepening their contemplation: “*I wanted to understand it, but it didn't speak to me. […] At first, I felt humiliated by the work. […] But then I saw that it wasn't laughing at me. I saw a laugh and I liked it. [….]*” (R14); “*I can't understand you if you are deafening me*” (R9).

##### 7.2.2.3 Emotional dimension

Regarding the emotional internalization of the experience of contemplating Maselli's work, some have referred to positive emotions that generate wellbeing: “*When looking at the mountains in the background, they conveyed hope to me […]*” (M6); “*I have felt tranquility, calm, silence*” (M12); “*I perceived a very deep silence, of peace, like a wellbeing*” (M15); “*I felt secure […] incredibly good […] I felt peace, enthusiasm, joy, and satisfaction*” (M18); hope: “*Looking at the distant mountains transmitted hope to me, behind those mountains there is something good*” (M6); awe (M26); wonder (M10).

Several participants mentioned negative or challenging emotions such as fear: “*[…] those rocky, dangerous peaks are frightening*” (M11); unease*: “it transmitted unease to me*” (M6, M25); anguish and anxiety: “*I felt a lot of anguish seeing my smallness reflected against the vastness […] I felt a kind of anxiety*” (M6).

Some participants felt contradictory emotions: “*I have felt nostalgia, but at the same time, peace, beauty, wonder*” (M10); “*I have been duality and contradiction. It has fascinated me*” (M14).

In the case of Rothko's work, the predominant emotions are those of wellbeing: “*The painting conveys emotional intensity, almost overflowing. Mainly it is the vibration of the dominant colors […] vitality*” (R1); “*It has conveyed joy and tranquility to me*” (R6); “*It conveys peace and calm with those lines in which I sense movement. It also inspires warmth*” (R13); “*It has made me feel secure, very happy*” (R19).

Negative or challenging emotions have also been mentioned: “*[It has been] very surprising*” (R4); “*I have felt small and alone and helpless before the scream*” (R9); “*It doesn't produce any kind of tranquility or calm for me at all, no! It seems to me too much passion*” (R25); as well as contradictory emotions: “*It has been pleasant […] at first, I felt unease […] I was curious to decipher the meaning*” (R12); “*It has been a mix of impressions, sensations, memories, internal conversations. It began by giving me a lot of peace [then] it overwhelmed me a bit*” (R20).

##### 7.2.2.4 Transcendent-spiritual dimension

In the case of Maselli, references to the transcendent-spiritual dimension appear: “*I have thought about my position in the world and my relationship with what surrounds me*” (M1); “*It is a very spiritual painting*” (M6); “*[…] it is a light that reminds you: God is here, you are protected, you are not abandoned*” (M11); “*I see the goal I want to reach*” (M13); “*I have rested and connected with nature and, consequently, with transcendence […] I have felt and understood the need to explain my presence in the world and resolve what pertains to the transcendence of my life*” (M26); “*Everything refers me to greatness, a greatness that complements the large with the small*” (M27).

In the case of Rothko: “*It is a completely spiritual painting, it speaks to us of essence, of the essence of the human being that is often disguised within each of us*” (R6), “*[It is an] invitation to transcendence, to faith, to hope*” (R22).

## 8 Discussion

According to the hypothesis the study shows:

H1. The contemplation of artworks by Fernando Maselli and Mark Rothko can provoke a significant aesthetic experience of the Sublime for HSP. Our starting point for this hypothesis is that the subject matter of a work of art is not a determining factor for it to become the trigger of an aesthetic experience that leads to the sublime (see the end of Section 1, with references to Stamatopoulou et al., 2024, and Fingerhut and Prinz, 2018).

The contemplative activity, designed according to Section 1, provoked relevant aesthetic experiences of the Sublime. The intensity of the Sublime aesthetic experience had a strong impact in both artworks, slightly higher in Rothko (8.4) than in Maselli (8.3). Responses to item C15, which specifically addresses the concept of the Sublime, indicate a higher score for Maselli (4.3) than for Rothko (3.3).

The qualitative results also support the relevance and depth of the aesthetic experience reported in both cases. As established in the literature discussed in introductory sections, in both artworks the Sublime was consistently associated with concepts such as astonishment (E1), fear (E2), joy (E3), unease (E11), admiration (E15), or infinity (C10). Thus, it can be said that contemplating the artworks of both Fernando Maselli and Mark Rothko may evoke a profound aesthetic experience of the Sublime in highly sensitive individuals; however, it is worth noting that in Maselli's case, explicit references to the Sublime are more frequent.

H2. The Sublime Experience Scale has a factorial structure that encompasses four dimensions (as said in Section 2) that, when integrated, show the intensity of the aesthetic experience with the Sublime.

The four dimensions were significantly valued, which means that all were activated to contribute to the aesthetic experience of the Sublime. As shown in Table 1, the values directly related to the Experience of the Sublime are high or very high for both Maselli and Rothko (ranging from 2.5 to 4.4).

The qualitative results also show the significance of all dimensions in both artworks (for example M10 and R26). In the perceptual dimension, both Maselli's and Rothko's works elicited references to sensory aspects such as temperature and nuances, which participants reported as becoming increasingly intense over the course of the contemplation. While Maselli's work prompted more frequent mentions of smell and sound, Rothko's evoked strong associations with taste.

In the cognitive dimension, both Maselli's and Rothko's works prompted deep personal reflections that went beyond the material presence of the artworks. More specifically, Maselli's landscapes often evoked symbolic readings and inner dialogue, leading some to reflect on personal growth or existential questions. In Rothko, the cognitive responses were frequently marked by intense imagery and metaphor, ranging from war and suffering to human connection. It is worth noting that, when facing Rothko's abstract painting, some participants described moments of profound insight, while others experienced difficulty accessing meaning, highlighting the complexity and variability of cognitive engagement in aesthetic experience.

In the emotional dimension, participants described both artworks as evoking a wide range of emotional responses, including positive, negative, challenging, and even contradictory feelings, sometimes within the same individual. It is remarkable the general appearance in two artworks of emotions such as astonishment (E1), fear (E2), joy (E3), unease (E11), admiration (E15). These emotions contribute to the generation of the aesthetic experience of the sublime (see Section 3). In the case of Maselli, emotions like astonishment (E1), fear (E2), unease (E11), and admiration (E15) predominate. In the case of Rothko, emotions related to wellbeing tended to predominate, although it also elicited feelings of aggression. In any case, the emotional engagement with the artworks is beyond question (for example R23, that highlights the “emotional weight”), even though, as will be seen below, the scores related to the emotional dimension are the lowest when compared to other dimensions.

In the transcendent-spiritual dimension, as the concept of sublime implies (see Section 3), Maselli's work elicited reflections on the contrast between human smallness and natural vastness, an invitation to continue seeking and discovering, a sense of personal positioning in the world in relation to the surrounding environment, and explicit references to God and divine protection. In contrast, spiritual references in responses to Rothko's work were less frequent, and centered around notions such as essence, the source of life, Truth, and Hope.

H3. Contemplating the Sublime, figurative art of Maselli affects the different spheres of a person more significantly than the abstract art of Rothko, and also the experience of the Sublime is better.

It is noteworthy that the values found for Maselli were higher than those recorded for Rothko in all spheres, highlighting, for example, that the lowest score was in the emotional sphere (3.18), followed by the cognitive (3.30) and perceptual (3.72) spheres, with the highest score being in the transcendental sphere (3.74). In the case of Rothko, the lowest score was also in the emotional sphere (2.72), followed by the cognitive (2.85) and transcendental (3.01) spheres, with the highest score being in the perceptual sphere (3.58).

Regarding the intensity of the experience associated with the impact on the four proposed dimensions, it is observed that although the values obtained in Maselli are higher in the personal spheres, the experience is more highly valued in the case of Rothko (3.7) compared to Maselli (3.6). This difference generally contrasts with the values obtained in the different dimensions, where Maselli's values are higher. For example, as previously mentioned, in item C15 of the cognitive sphere, which specifically asks about the Sublime, there is a significant difference between Maselli (4.2) and Rothko (3.3). This also occurs in items from different spheres, as shown in Table 1, where all the values for Maselli exceed those for Rothko, except in item E11, where they have the same value (3.1). This apparent contradiction may be due to the fact that Rothko's work promotes more unconscious internal stimuli, which are not reflected in the questionnaire through rational questions but do affect the experience according to Didi-Huberman (2006). In the case of Maselli, the stimuli he promotes are more external, in the sense that what he shows is more recognizable and closer to our knowledge of the world. So much so that item E19, which captures immersion in the artwork, has a higher value for Maselli (4.3) than for Rothko (3.8), as was to be expected. Indeed, in response to Maselli's work, some participants reported feeling “as if inside a film,” experiencing tactile and auditory sensations. In this vein, they referenced works, films, or places such as *The Little Prince, Society of the Snow*, or Vinicunca (Mountain of Seven Colors in Peru). In contrast, Rothko's work more frequently prompted a connection with the artist, the technique, or interpretation itself, fostering an external and dialogical connection within the group.

Furthermore, the following points should be highlighted. The most prominent material features of the artworks were texture and color. While Maselli's work emphasized contrast and the absence of color, Rothko's generated controversy around the essential color of the piece. In both cases, the contemplation led to a deeper exploration of the participants' own conception of space and time. In Maselli's work, more unconscious internal stimuli were activated, allowing participants to perceive immaterial aspects that touched their deeper sense of self. In Rothko, although such internal stimuli were also abundant, some participants reported not having accessed the depth of the work or feeling particularly addressed by it. Thus, Maselli's artwork facilitated a sense of communication with the piece, whereas Rothko's left some participants struggling to engage more deeply with the artwork.

## 9 Conclusions

Regarding hypothesis HP1, the results from the open-ended questions of the “Sublime Experience Scale” confirm that both the contemplation of the works of Maselli and Rothko produce an aesthetic experience of the sublime, and that reaches the perceptual, cognitive, emotional, and transcendent-spiritual dimensions of the Highly Sensitive Person.

Regarding hypothesis HP2, The designed evaluation instrument serves as a measurement guide for the Sublime aesthetic experience, taking into account the different personal spheres in an integrated manner. The “Sublime Experience Scale” is configured as a measurement instrument that captures relevant aspects of the aesthetic experience of the sublime in four dimensions: perceptual, emotional, cognitive, and transcendent. All participants showed high scores across all spheres, indicating an integrated experience.

Regarding hypothesis HP3, the figurative style of Maselli allows for a more intense impact on the perceptual, emotional, cognitive, and transcendental spheres of Highly Sensitive Individuals. However, the intensity of the aesthetic experience related to the Sublime is slightly greater in the abstract works of Rothko. This may have been caused by the limitations of the measurement instrument, which was unable to capture unconscious aspects that the contemplation of Rothko's work may provoke. The Sublime scale was agreed upon by all the authors but was not tested with a pilot group. Only the original scale by Urpí et al.'s (2022), had been validated at the time. This aspect could have been addressed to strengthen the study.

Furthermore, the sample was small, consisting of only 27 participants, so it could be interesting to expand it, exploring the possibility of including individuals who are not Highly Sensitive Persons. In that respect, the contribution of this study may lie in establishing a starting point toward that goal. In fact, thinking about future continuity in this research, we wonder whether this experience can be transferred to other groups, to other museums, and to other figurative or abstract works that allow for provoking experiences related to the Sublime. The main contribution of this work is to offer a foundation for further group experiences and the advancement of additional theory on the topic.

## Data Availability

The raw data supporting the conclusions of this article will be made available by the authors, without undue reservation.
