# Correction: The aesthetic experience of the sublime for a group of Highly Sensitive Persons: Maselli's figurative style vs. Rothko's abstract expressionism

**DOI:** 10.3389/fpsyg.2026.2016263

**Published:** 2026-10-16

**Authors:** Fernando Echarri, Ignacio Miguéliz, Natalia Verea, Teresa Barrio

**Affiliations:** 1 University of Navarra Museum, Pamplona, Spain; 2 Education and Psychology Faculty, University of Navarra Museum, Pamplona, Spain

**Keywords:** sublime, contemporary art, contemplation, aesthetic experience, emotions, Highly Sensitive Person

The supplemental material that has been published is incorrect. It is requested to remove the supplemental material.

There was missing information about the preliminary validation.

A correction has been made to the *Section 6.3 Instrument proposal*, first paragraph:

“To evaluate the experience, the Scale for Evaluating Aesthetic Experience developed by Urpí et al. (2022), which has undergone preliminary validation, was adapted to include items more related to the attributes of the concept of the Sublime gathered from the literature. That is why it was called the “Sublime scale.” The adaptation of this measurement tool was debated and jointly agreed upon by the study's researchers, with an awareness of the limitations involved in gathering reliable data based on participants' self-reports (Fingerhut and Prinz, 2018, p. 122).”

The original version of this article has been updated.

