# Supplementary material for: The aesthetic experience of the sublime for a group of Highly Sensitive Persons: Maselli's figurative style vs. Rothko's abstract expressionism
Source: Front Psychol. 2025 Sep 17;16:1609994. doi: 10.3389/fpsyg.2025.1609994 (PMC12486599; doi:10.3389/fpsyg.2025.1609994)
Supplement: Supplementary file 1 [file Data_Sheet_1.pdf]

Escala de valoración de la Experiencia Estética

Adaptada de C. Urpi, Ch. Reparaz, F. Echarri y C. Iriarte

Sexo:
Edad:
Obra de arte contemplada:

INSTRUCCIONES

Por favor, responde a las siguientes preguntas marcando la casilla elegida con una X

Es muy importante que respondas a todas las cuestiones.
Muchas gracias

**1.**	**Dentro del cuadro, mientras lo miro noto:**	**Nada**<br><br>**1**	**Casi nada**<br>**2**	**Poco**<br><br>**3**	**Bastante**<br><br>**4**	**Mucho**<br><br>**5**
P1	Profundidad espacial					
P2	Luces y sombras					
P3	Texturas (sensaciones si lo pudiera tocar)					
P4	Movimiento en las formas del cuadro					
P5	Colores que cambian					
P6	Sonidos dentro del cuadro					
P7	Olores dentro del cuadro					
P8	Las temperaturas de los colores					
P9	Sutileza (detalles sutiles)					

**2.**	**El cuadro me hace sentir:**	**Nada**<br><br>**1**	**Casi nada**<br>**2**	**Poco**<br><br>**3**	**Bastante**<br><br>**4**	**Mucho**<br><br>**5**
E1	Asombro					
E2	Miedo / Temor					
E3	Alegría					
E4	Horror					
E5	Tristeza					
E6	Enfado					
E7	Interés					
E8	Asco					
E9	Relajación / Tranquilidad					
E10	Atracción / Placer					
E11	Inquietud					
E12	Bienestar					
E13	Aburrimiento					

E14	Entusiasmo					
E15	Admiración					
E16	Grandeza					
E17	Soledad					
E18	Melancolía					
E19	Inmersión en la obra					
E20	Abrumado					
E21	Inmensidad					
E22	Curiosidad					

**3.**	**El cuadro me hace pensar en:**	**Nada**<br><br>**1**	**Casi nada**<br>**2**	**Poco**<br><br>**3**	**Bastante**<br><br>**4**	**Mucho**<br><br>**5**
C1	Experiencias que me han pasado					
C2	Personas reales o imaginarias					
C3	Lugares reales o imaginarios					
C4	Nuevas ideas					
C5	Lo que representa o significa					
C6	Otras obras de arte					
C7	Los motivos del artista para crearlo					
C8	Pintar yo algo parecido					
C9	Su valor artístico					
C10	Lo infinito					
C11	Lo inalcanzable					
C12	Mi insignificancia, pequeñez					
C13	Mi lugar en el mundo					
C14	La belleza material					
C15	Lo sublime					

**4.**	**Cuando estoy delante del cuadro:**	**Nada**<br>**1**	**Casi nada**<br>**2**	**Poco**<br>**3**	**Bastante**<br>**4**	**Mucho**<br>**5**
R1	Quiero estar solo, desconectar del mundo					
R2	Quiero compartirlo con otras personas					
R3	Quiero rezar/meditar					
R4	Pienso en el sentido de la vida					
R5	Pienso en mi propia existencia					
R6	Pienso en la trascendencia después de la muerte, el más allá					
R7	Siento que Dios existe					
R8	Descubro la belleza trascendente					

5.	**Contemplar la obra de arte ha sido una experiencia que:**	**Nada**<br><br>**1**	**Casi nada**<br>**2**	**Poco**<br><br>**3**	**Bastante**<br><br>**4**	**Mucho**<br><br>**5**
S1	Me ha encantado					
S2	Me gustaría contar a otras personas					
S3	Ha sido valiosa para mi vida					
S4	Ha hecho que se me pase el tiempo rápidamente					
S5	Ha hecho que me olvide de dónde estoy					
S6	Me ha servido para comprender mejor el arte contemporáneo					
S7	Recordaré mucho tiempo					
S8	Me gustaría repetir					
S9	Me ha conmovido profundamente					

6. ¿Podrías valorar tu satisfacción con la experiencia de contemplación de la obra de arte de 0 a 10?

**1**	**2**	**3**	**4**	**5**	**6**	**7**	**8**	**9**	**10**

7. ¿Podrías describir con tus palabras cómo ha sido tu experiencia de contemplación de la obra? Tienes todo el espacio que necesites para escribir.

....
....
....
....
....
....
....
....
....
....
....
....
....
....
....
....
....
....
